# Neuronal Cell‐Type Susceptibility in the Ventral Subiculum of 5xFAD Mice

**DOI:** 10.1002/alz.089493

**Published:** 2025-01-03

**Authors:** Maricarmen Pachicano, Neda Khanjani, Angela Hurtado, Shrey Mehta, Zion MK Smith, Michael S Bienkowski

**Affiliations:** ^1^ Stevens Neuroimaging and Informatics Institute, Keck School of Medicine, University of Southern California, Los Angeles, CA USA; ^2^ USC Center for Integrative Connectomics, Los Angeles, CA USA

## Abstract

**Background:**

Identification of cell‐type vulnerability in Alzheimer’s Disease (AD) is critical to the clinical development of targeted treatments. Neurodegeneration of the subiculum (SUB) is an early biomarker of AD, but it is unknown if specific SUB cell‐types are susceptible to AD neurodegeneration. In the 5xFAD mouse model, significant cell loss occurs within the SUB by 8 months of age. Our previous work creating the mouse Hippocampus Gene Expression Atlas (HGEA) has defined cell‐type specific gene markers that can identify cell‐types within the SUB (Bienkowski et al, 2018). Within the ventral SUB (SUBv), three relatively discrete layers of gene expression define cell‐type organization. Quantification of SUBv cells identified by these gene markers can be utilized to determine SUB cell‐type susceptibility across disease timepoints.

**Method:**

To label SUBv gene expression patterns, we used RNAscope single molecule fluorescent in situ hybridization (smFISH) to label and quantify individual RNA transcript levels within SUB cell types. 5xFAD and wildtype (WT) mice (2, 6, 12, and 14 mo.) were perfused, dissected, and coronally sectioned into 20µm thick sections. SUBv tissue sections were hybridized with probes designed to target SUBv gene expression markers using RNAscope Multiplex Fluorescent V2 kit (Advanced Cell Diagnostics). Tissue sections were counterstained for DAPI cytoarchitecture, imaged at 40X with a spinning disk confocal microscope, and quantification was performed using QuPath (Quantitative Pathology and Bioimage Analysis) cell segmentation and SCAMPR analysis software (Ghoddousi et al, 2022).

**Result:**

RNAscope labeling produced robust datasets enabling quantification of single RNA transcript levels for each gene expression marker within 10,000+ SUBv cells. At late disease timepoints, we found that the number of SUBv layer 3 cells were significantly diminished compared to WT controls suggesting that SUBv layer 3 cells are primarily susceptible to AD neurodegeneration. No significant difference was found between 5xFAD and WT mice at 2 mo. of age.

**Conclusion:**

In the SUBv, layer 3 neurons are primarily susceptible to AD neurodegeneration in the 5xFAD mouse model. Future studies will investigate the transcriptomic changes that occur within SUBv layer 3 neurons that precede death.

